# p70 S6 kinase drives ovarian cancer metastasis through multicellular spheroid-peritoneum interaction and P-cadherin/β1 integrin signaling activation

**DOI:** 10.18632/oncotarget.2362

**Published:** 2014-08-21

**Authors:** Carman Ka Man Ip, Susan Yung, Tak-Mao Chan, Sai-Wah Tsao, Alice Sze Tsai Wong

**Affiliations:** ^1^ School of Biological Sciences, University of Hong Kong, Pokfulam Road, Hong Kong; ^2^ Department of Medicine, University of Hong Kong, Sassoon Road, Hong Kong; ^3^ Department of Anatomy, University of Hong Kong, Sassoon Road, Hong Kong

**Keywords:** p70^S6K^, P-cadherin, β1 integrin, adhesion, metastasis

## Abstract

Peritoneal dissemination as a manifestation of ovarian cancer is an adverse prognostic factor associated with poor clinical outcome, and is thus a potentially promising target for improved treatment. Sphere forming cells (multicellular spheroids) present in malignant ascites of patients with ovarian cancer represent a major impediment to effective treatment. p70 S6 kinase (p70^S6K^), which is a downstream effector of mammalian target of rapamycin, is frequently hyperactivated in human ovarian cancer. Here, we identified p70^S6K^ as an important regulator for the seeding and successful colonization of ovarian cancer spheroids on the peritoneum. Furthermore, we provided evidence for the existence of a novel crosstalk between P-cadherin and β1 integrin, which was crucial for the high degree of specificity in cell adhesion. In particular, we demonstrated that the upregulation of mature β1 integrin occurred as a consequence of P-cadherin expression through the induction of the Golgi glycosyltransferase, ST6Gal-I, which mediated β1 integrin hypersialylation. Loss of p70^S6K^ or targeting the P-cadherin/β1-integrin interplay could significantly attenuate the metastatic spread onto the peritoneum *in vivo*. These findings establish a new role for p70^S6K^ in tumor spheroid-mesothelium communication in ovarian cancer and provide a preclinical rationale for targeting p70^S6K^ as a new avenue for microenvironment-based therapeutic strategy.

## INTRODUCTION

With approximately 200,000 new cases, and more than 125,000 related deaths occur each year worldwide, ovarian cancer is the most lethal of all gynecologic cancers [1]. The high rate of deaths is due to the large tumor burden with extensive peritoneal metastatic lesions. Despite initial chemosensitivity, ovarian cancer recurrence or peritoneal metastasis results in poor prognosis (<25%) and represents a serious clinical challenge. While it is known that localized peritoneal dissemination predominates over hematogenous or lymphatic spread, the exact mechanisms are still unknown. Successful adhesion on the peritoneal mesothelium represents a key rate-limiting step for the onset of the metastatic cascade. Unraveling the underlying molecular pathways is critical for developing new therapeutic strategies.

Emerging basic research, preclinical and clinical findings support the importance of p70 S6 kinase (p70^S6K^), a downstream effector of mammalian target of rapamycin (mTOR), in the progression of ovarian cancer [2]. Indeed, activation of mTOR is almost ubiquitous in ovarian carcinoma lesions and often correlates with poor prognosis [3]. p70^S6K^ activation can result from the enhanced expression and activity of cytokines and growth factors, such as hepatocyte growth factor (HGF) that characterizes ovarian cancer [4]. p70^S6K^ is activated more often in high-grade ovarian carcinomas, which highlights the need to understand the role of p70^S6K^ in ovarian cancer and disease progression [5]. Although the involvement of p70^S6K^ in the control of growth has been well established, our recent studies showed for the first time that p70^S6K^ could be involved in other aspects of tumor progression such as metastasis [4, 6]. However, we do not know if p70^S6K^ plays a role during peritoneal adhesion and dissemination, and if so, how it regulates this process.

Cells propagated to form multicellular spheroids mimic most of the in vivo properties of tumors [7]. This is particularly relevant for ovarian cancer in view of the fact that tumor spheres commonly found in malignant ascites of ovarian cancer patients represent a significant impediment to efficacious treatment [8]. The observation that cancer stem/tumor-initiating cells can also be enriched by spheroids in suspension cultures further suggest spheroid cells may be associated with tumor aggressiveness [9]. Thus, cells cultured as 3D spheroids can be used as a tool to evaluate key metastatic events in the cascade.

Cadherins and integrins are major classes of cell surface receptors that mediate cell-cell and cell-matrix adhesion, respectively [7]. Cadherins, especially E-, N-, and P-cadherin have been implicated in the pathogenesis of ovarian cancer [8], and several integrins are expressed by ovarian tumor cells such as α2β1, α5β1, and αvβ3 [9]. However, the mechanistic basis of their crosstalk in ovarian cancer is not completely understood.

In this study, we show for the first time a role for p70^S6K^ in the adhesion and metastatic spread of highly malignant ovarian cancer spheroids into the peritoneum. We also present evidence that a novel interplay linking P-cadherin to regulation of β1 integrin activation via the induction of the Golgi glycosyltransferase, ST6Gal-I, which mediates β1 integrin hypersialylation in this process.

## RESULTS

### p70^S6K^ promotes ovarian cancer spheroid adhesion to peritoneal mesothelium

**Figure 1:**
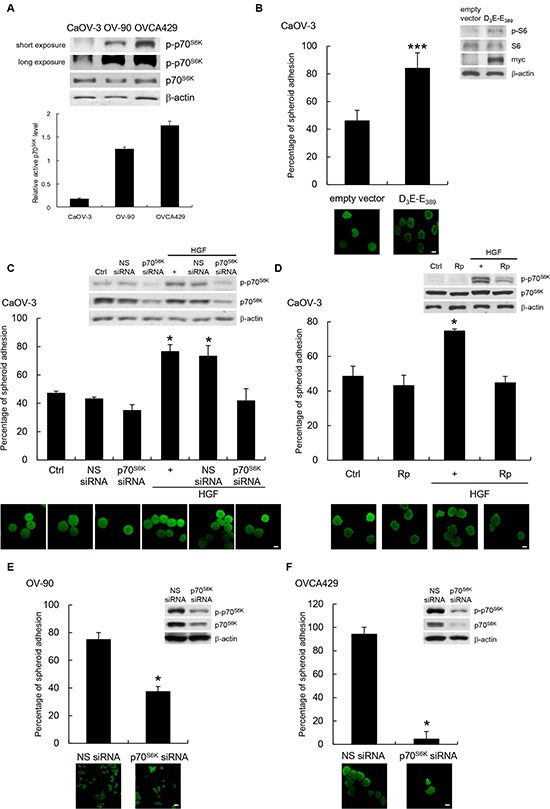
p70^S6K^ activation promotes cancer spheroid adhesion to human peritoneal mesothelial cells **(A)** Whole cell lysates were analyzed for levels of phosphorylated (p-) and total p70^S6K^ by Western blotting with β-actin was as the loading control. Signal intensity was determined by densitometry, and the level of p-p70^S6K^ was normalized against total p70^S6K^. **(B)** Fluorescent-labeled CaOV-3 spheroids transfected with empty vector or myc-tagged constitutively active p70^S6K^ (D_3_E-E_389_) were plated onto a confluent mesothelium monolayer and allowed to adhere for 5 h. The percentage of adherent spheroids was quantified after nonadherent spheroids were removed. Whole cell lysates were analyzed for levels of phosphorylated (p-) and total S6 and myc by Western blotting (inset). CaOV-3 spheroids **(C)** expressing nonspecific (NS) or p70^S6K^ siRNA or **(D)** pretreated with rapamycin (Rp; 20 nM) before stimulation with hepatocyte growth factor (HGF; 10 ng/ml) for 16 h were harvested for the adhesion assay with mesothelial cells. The activities of p70^S6K^ were analyzed by Western blotting (inset). **(E)** OV-90 or **(F)** OVCA429 spheroids transfected with NS or p70^S6K^ siRNA were used in the adhesion assay with mesothelial cells. The knockdown efficiencies of p70^S6K^ were analyzed by Western blotting (inset). Data are expressed as mean ± S.D. *, *P* < 0.05; ***, *P* < 0.001 vs. control, empty vector or NS siRNA expressing spheroids.

The first step in ovarian cancer metastasis is adhesion of cancer cells on the peritoneum. To investigate if p70^S6K^ plays a role in influencing the adhesion of ovarian cancer cells to the peritoneum, we used a fluorescent-based coculture assay to monitor the interactions between tumor spheroids and primary human mesothelial cells obtained from patients to model the *in vivo* condition of ovarian cancer. We first examined the intracellular level of p70^S6K^ by Western blot analysis in three different human ovarian cancer cell lines (CaOV-3, OV-90, and OVCA429) (Figure 1A). CaOV-3 cells showed little expression of active p70^S6K^, whereas OV-90 and OVCA429 showed strong expression of active p70^S6K^. We then transfected constitutively activated p70^S6K^ (myc-tagged D_3_E-E_398_) into CaOV-3 cells. Western blot analysis revealed that D_3_E-E_398_ increased p70^S6K^ activity, as indicated by an increase of phosphorylation of S6, a substrate of p70^S6K^ (Figure 1B, inset). Importantly, ectopic expression of D_3_E-E_398_ significantly increased adhesion of CaOV-3 spheroids to the mesothelial monolayer (Figure 1B). To confirm the importance of p70^S6K^ in modulating mesothelial adhesion, we employed a small interfering RNA (siRNA) specifically engineered towards p70^S6K^. In these studies, HGF was used to activate p70^S6K^, because HGF is highly expressed in ascitic fluid of ovarian cancer patients, marking it as a major contributor to malignant spreading [10]. We and others have shown that Met tyrosine kinase, a high affinity receptor for HGF, is often overexpressed in ovarian carcinomas and cancer cell lines [4, 11], and constitutive activation of the Met receptor through paracrine/autocrine mechanisms has been observed with progressive neoplastic changes [11-13]. The effectiveness of p70^S6K^-specific siRNA, but not nonspecific siRNA, to deplete p70^S6K^ expression was confirmed by Western blotting (Figure 1C, inset). Transfection of p70^S6K^ siRNA in Met-expressing CaOV-3 spheroids inhibited the HGF-mediated adhesion to mesothelial cells (44% inhibition, *P* = 0.02) (Figure 1C) [4, 11]. We validated these results using an independent siRNA sequence targeting p70^S6K^. This effect was similar to the inhibition of cancer spheroid adhesion by rapamycin (40% inhibition, *P* = 0.041), a small molecule inhibitor of p70^S6K^ kinase activity (Figure 1D). To complement our studies, we used two other independent cell lines, OV-90 and OVCA429, which have high basal p70^S6K^ activities. Spheroid cells treated with p70^S6K^ siRNA resulted in substantially decreased p70^S6K^ phosphorylation (OV-90: 75% inhibition; OVCA429: 86% inhibition) and p70^S6K^ expression (OV-90: 72% inhibition; OVCA429: 93% inhibition) (Figure 1E & 1F, inset). The siRNA treated OV-90 and OVCA429 spheroids with reduced p70^S6K^ levels were incapable of effectively attaching to mesothelial cells, whereas nonspecific siRNA treated spheroids were unaffected (Figure 1E & 1F). These data suggest that p70^S6K^ is an important mediator of ovarian cancer spheroid adhesion to the peritoneal mesothelium.

### p70^S6K^ activation enhances P-cadherin and β1 integrin expression

**Figure 2:**
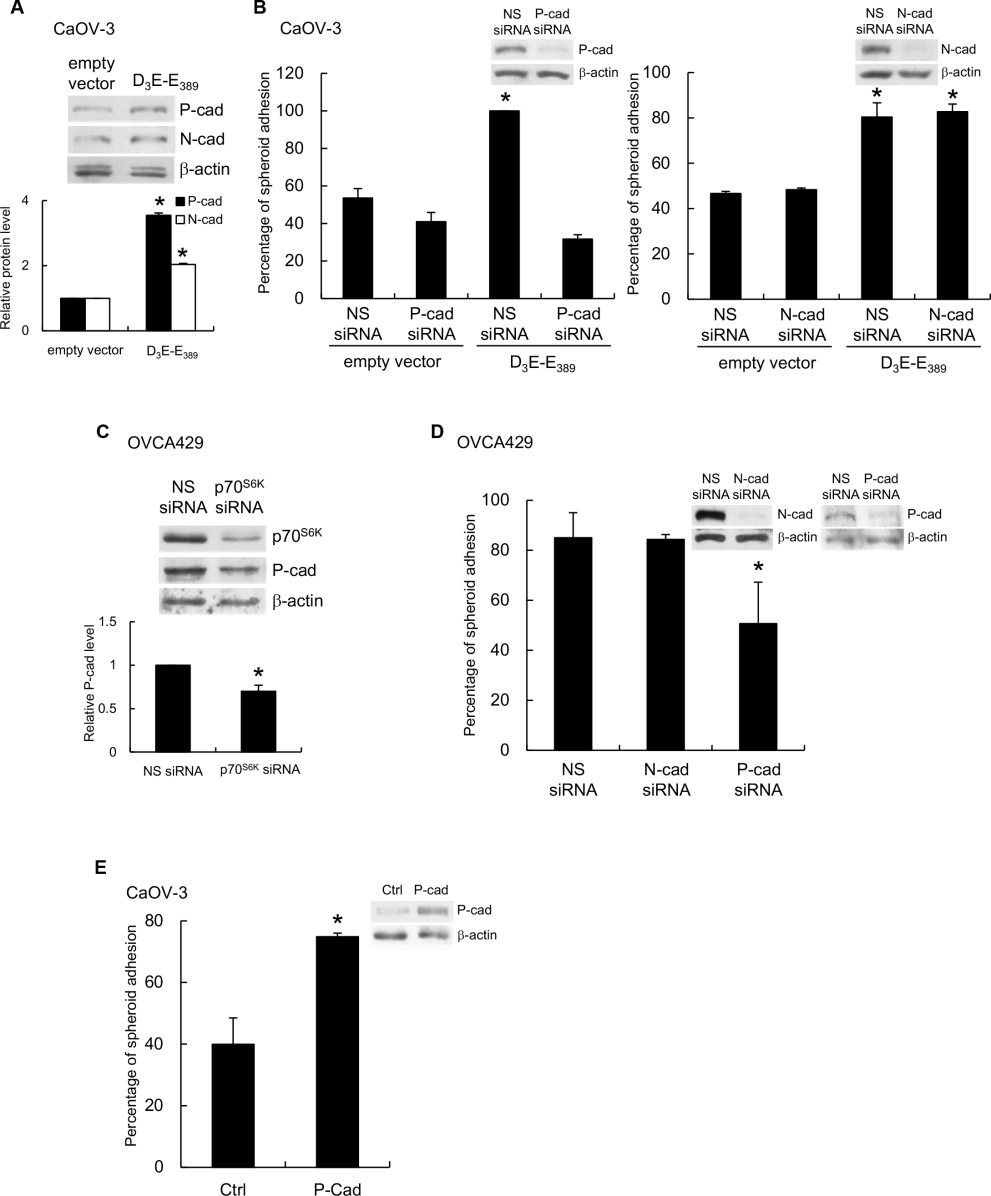
P-cadherin mediates p70^S6K^-dependent spheroid-mesothelium adhesion **(A)** Whole cell lysates of CaOV-3 spheroids transfected with empty vector or myc-tagged constitutively active p70^S6K^ (D_3_E-E_389_) were analyzed for levels of N- and P-cadherin by Western blotting with β-actin as the loading control. The signal intensity was determined by densitometry, and levels of N- and P-cadherin were normalized against the β-actin control. **(B)** Spheroids cotransfected with empty vector or myc-tagged D_3_E-E_389_ and nonspecific (NS) siRNA, P-cadherin siRNA (left) or N-cadherin siRNA (right) were plated onto a confluent mesothelium monolayer and allowed to adhere for 5 h. The percentage of adherent spheroids was quantified after nonadherent spheroids were removed. **(C)** The levels of p70^S6K^ and P-cadherin in OVCA429 spheroids transfected with NS siRNA or p70^S6K^ siRNA were analyzed by Western blotting. **(D)** OVCA429 spheroids transfected with NS siRNA, N-cadherin siRNA, or P-cadherin siRNA, and **(E)** CaOV-3 spheroids stably expressing P-cadherin were harvested for the adhesion assay with mesothelial cells. The expression of P-cadherin was analyzed by Western blotting. Data are expressed as mean ± S.D. *, *P* < 0.05 vs. control, empty vector or NS siRNA expressing spheroids.

To address the mechanism by which p70^S6K^ signaling could influence cellular adhesion to the mesothelium, we investigated adhesion receptors peritoneal environment as a possible mechanism. We first determined whether p70^S6K^ activation affected the expression of N- and P-cadherin present in the ovarian tumor/peritoneal microenvironment [8]. Although D_3_E-E_398_-expressing CaOV-3 showed increased expression of both N- and P-cadherin (Figure 2A), depletion of P-cadherin (Figure 2B, left), but not N-cadherin (Figure 2B, right), resulted in decreased adhesion of p70^S6K^-expressing cancer spheroids to the mesothelium, whereas treatment with nonspecific siRNA had no effect (Figure 2B). In addition, depletion of p70^S6K^ in OVCA429 spheroids caused significant decreased P-cadherin expression and inhibited their adhesion to the mesothelium (Figure 2C & 2D). We also showed that spheroid-mesothelium adhesion was significantly increased in P-cadherin-overexpressing spheroids compared to control spheroids (~2-fold, *P* = 0.011), indicating this process was P-cadherin dependent (Figure 2E).

**Figure 3:**
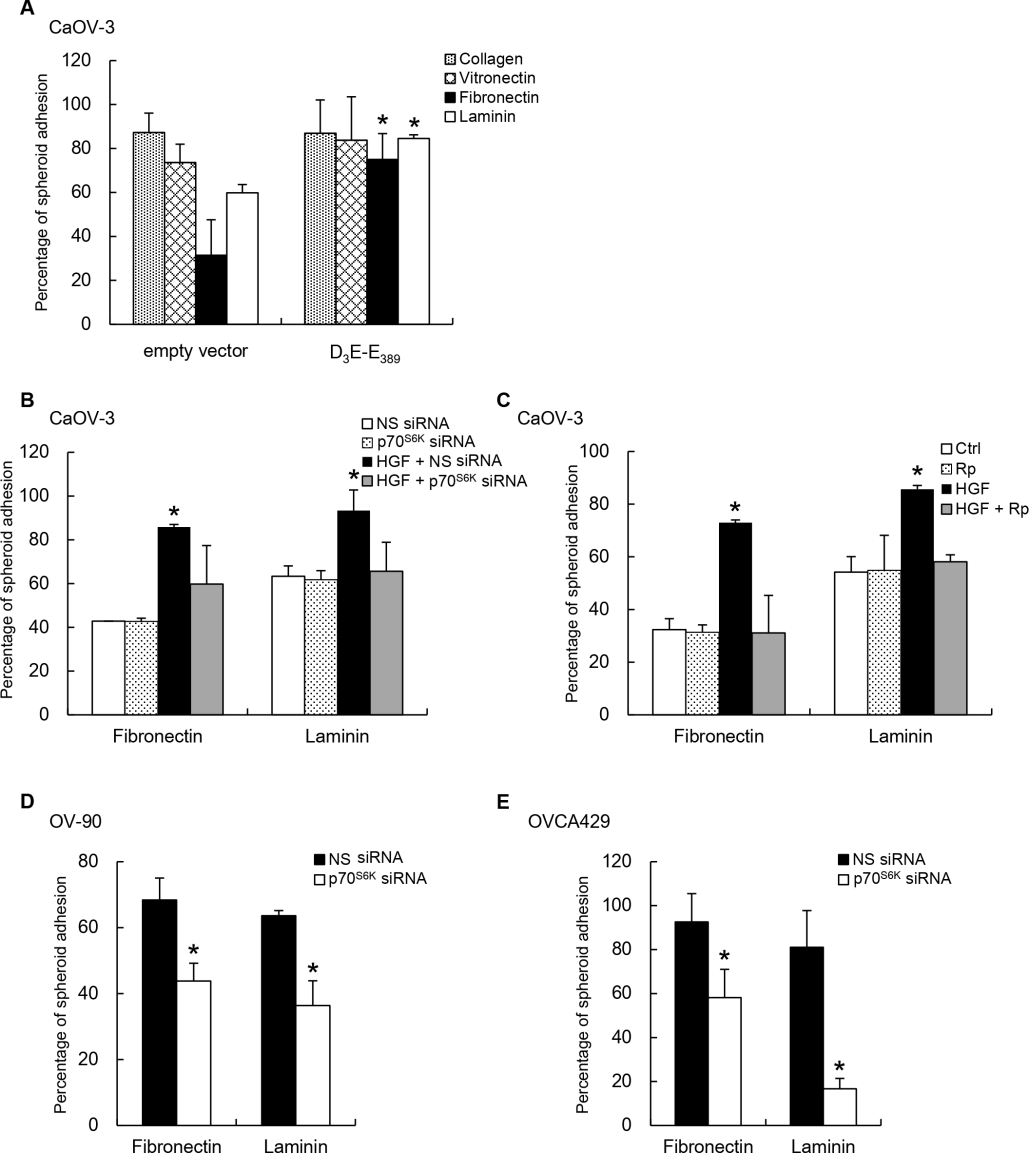
p70^S6K^ activation promotes spheroid adhesion to fibronectin and laminin **(A)** CaOV-3 spheroids expressing empty vector or constitutively active p70^S6K^ (D_3_E-E_389_) plasmid added to plates coated with collagen I, vitronectin, fibronectin, or laminin (10 μg/ml) for 2-5 h. **(B)** CaOV-3 cells expressing nonspecific (NS) siRNA or p70^S6K^ siRNA or **(C)** pretreated with rapamycin (Rp; 20 nM) before stimulation with hepatocyte growth factor (HGF; 10 ng/ml) were used in the adhesion assay with fibronectin or laminin. **(D)** OV-90 or **(E)** OVCA429 spheroids transfected with NS siRNA or p70^S6K^ siRNA were used in the adhesion assay with fibronectin or laminin. The percentage of adherent spheroids was quantified after nonadherent spheroids were removed. Data are expressed as mean ± S.D. *, *P* < 0.05 vs. control, empty vector or NS siRNA expressing spheroids.

The extracellular matrix (ECM) underlying the mesothelium lies on an (ECM) rich in fibronectin, type I collagen, laminin, and vitronectin [14, 15]. Given the effect of p70^S6K^ on adhesion to the mesothelium, we set out to assess if p70^S6K^ could also affect adhesion of cancer spheroids to different ECM components in the peritoneal environment. Compared to empty vector expressing control spheroids, D_3_E-E_398_-expressing CaOV-3 spheroids had significantly enhanced adhesion to fibronectin (2.4-fold, *P* = 0.022) and laminin (1.4-fold; *P* = 0.049), but not to collagen I or vitronectin (Figure 3A). However, CaOV-3 spheroids treated with p70^S6K^ siRNA almost completely inhibited HGF-mediated adhesion to fibronectin and laminin (Figure 3B), whereas treatment with nonspecific siRNA had no effect (Figure 3B). Furthermore, treatment with rapamycin also resulted in decreased spheroid attachment onto fibronectin and laminin (Figure 3C). In comparison, OV-90 and OVCA429 spheroids treated wtih p70^S6K^ siRNA also showed marked decreases in adhesion to fibronectin (OV-90: 36% inhibition, *P* = 0.012; OVCA429: 36% inhibition, *P* = 0.045) and laminin (OV-90: 43% inhibition, *P* = 0.049; OVCA429: 80% inhibition, *P* = 0.041), whereas treatment with nonspecific siRNA had no effect (Figure 3D & 3E).

**Figure 4:**
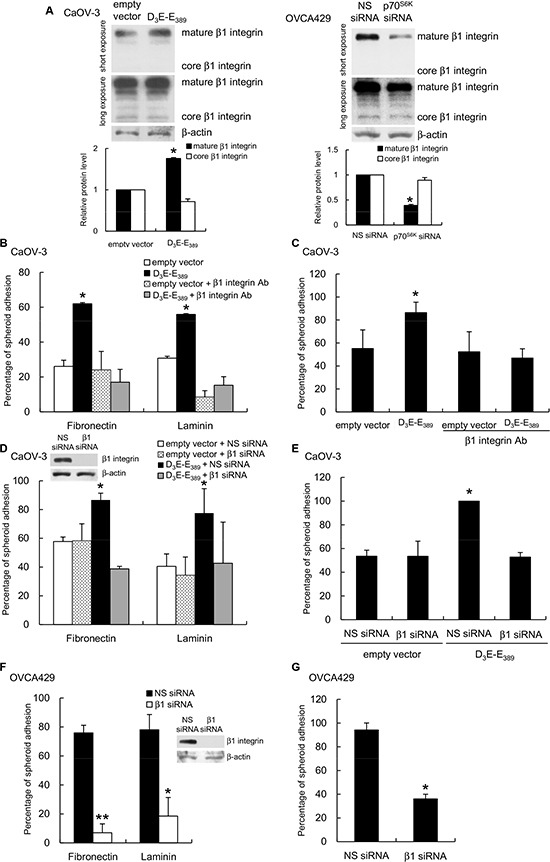
p70^S6K^ regulates β1 integrin expression and regulates cancer spheroid-ECM adhesion **(A)** Whole cell lysates of CaOV-3 spheroids transfected with empty vector or myc-tagged constitutively active p70^S6K^ (D_3_E-E_389_) were analyzed for core or mature form of β1 integrin expression by Western blotting with β-actin as the loading control. The signal intensity was determined by densitometry and levels of the core or mature form of β1 integrin were normalized against the β-actin control. **(B)** Spheroids were preincubated with β1 integrin neutralizing antibody (β1 integrin Ab) for the adhesion assay with fibronectin, laminin or **(C)** mesothelial cells. CaOV-3 spheroids cotransfected with empty vector or D_3_E-E_389_ and nonspecific (NS) siRNA or β1 integrin siRNA (β1 siRNA) were used in the adhesion assay with **(D)** fibronectin, laminin or **(E)** mesothelial cells. OVCA429 spheroids transfected with NS siRNA or β1 integrin siRNA were used in the adhesion assay with **(F)** fibronectin, laminin or **(G)** mesothelial cells. The knockdown efficiencies of β1 integrin were evaluated by Western blotting (inset). The percentage of adherent spheroids was quantified after nonadherent spheroids were removed. Data are expressed as mean ± S.D. *, *P* < 0.05; **, *P* < 0.005 vs. empty vector or NS siRNA expressing spheroids.

β1 integrin is known to be relevant in the process of adhesion to fibronectin and laminin [16]. We found β1 integrin expression was significantly enhanced in D_3_E-E_398_-expressing CaOV-3 spheroids (Figure 4A, left), whereas knockdown of p70^S6K^ resulted in decreased β1 integrin expression (Figure 4A, right). Interestingly, these changes in expression occurred in the 130 kDa mature form of β1 integrin, which represents the fully glycosylated, functional receptor, but no changes occurred in the ~85-kDa core peptide (Figure 4A). To test the specific involvement of the mature form of β1 integrin more directly, we treated D_3_E-E_398_-expressing CaOV-3 spheroids with blocking antibodies against β1 integrin. The inhibition of surface β1 integrin specifically inhibited the adhesion of spheroids to fibronectin, laminin (Figure 4B), and primary human mesothelial cells (Figure 4C). The inhibition of β1 integrin by specific siRNA treatment also inhibited 70^S6K^-dependent tumor spheroid attachment to fibronectin, laminin (Figure 4D), and mesothelial cells (Figure 4E). Similarly, β1 integrin siRNA-transfected OVCA429 showed significant reduction of spheroid adhesion to fibronectin, laminin (Figure 4F), and mesothelial cells (Figure 4G), which confirms the involvement of mature β1 integrin in ovarian cancer spheroid-mesothelium adhesion.

### P-cadherin functions upstream of β1 integrin in p70^S6K^ signaling

**Figure 5:**
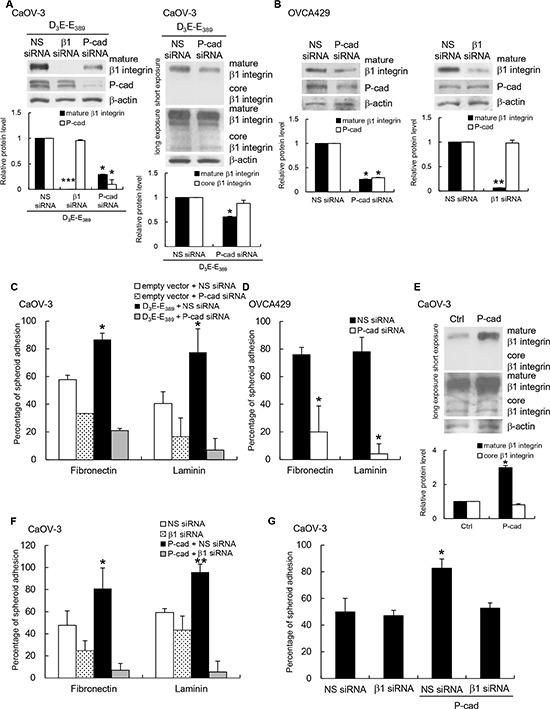
P-cadherin acts upstream of β1 integrin to mediate p70^S6K^-dependent adhesion **(A)** CaOV-3 spheroids cotransfected with constitutively active p70^S6K^ (D_3_E-E_389_) and nonspecific (NS) siRNA, β1 integrin siRNA (β1 siRNA), or P-cadherin siRNA, and **(B)** OVCA429 spheroids transfected NS siRNA, β1 integrin siRNA, or P-cadherin siRNA were analyzed for core or mature forms of β1 integrin and P-cadherin by Western blotting with β-actin as the loading control. The signal intensity was determined by densitometry, and levels of P-cadherin and the core or mature forms of β1 integrin were normalized against the β-actin control. **(C)** D_3_E-E_389_-expressing CaOV-3 or **(D)** OVCA429 spheroids transfected with NS siRNA or P-cadherin siRNA were used in the adhesion assay with fibronectin or laminin. **(E)** CaOV-3 stably expressing P-cadherin were analyzed for core or mature forms of β1 integrin by Western blotting with β-actin as the loading control. P-cadherin-expressing CaOV-3 transfected with NS siRNA or β1 integrin siRNA were collected for the adhesion assay with **(F)** fibronectin, laminin or **(G)** mesothelial cells. The percentage of adherent spheroids was quantified after nonadherent spheroids were removed. Data are expressed as mean ± S.D. *, P < 0.05; **, P < 0.005; ***, P < 0.001 vs. empty vector or NS siRNA expressing spheroids.

To investigate the possible crosstalk between P-cadherin and β1 integrin and to determine their interdependency, we examined the effect of P-cadherin and β1 integrin siRNAs on the expression of the two adhesion receptors in D_3_E-E_389_-expressing CaOV-3 spheroids. Knocking down β1 integrin did not affect P-cadherin expression (Figure 5A). However, depletion of P-cadherin did affect β1 integrin expression (Figure 5A), but only the expression of the mature β1 integrin was inhibited and not core β1 integrin (Figure 5A). For comparison, we also used OVCA429 spheroids, which gave us similar results (Figure 5B). Accordingly, treatment of CaOV-3 spheroids with P-cadherin siRNA abolished D_3_E-E_389_-mediated adhesion to fibronectin and laminin (Figure 5C), and again similar results were observed with OVCA429 spheroids (Figure 5D). Ectopic expression of P-cadherin significantly increased the levels of the mature β1 integrin, but not core β1 integrin (Figure 5E). After transfection with β1 integrin siRNA, P-cadherin-overexpressing CaOV-3 spheroids were unable to adhere to fibronectin, laminin (Figure 5F), and mesothelial cells (Figure 5G). The nonspecific siRNA treatment had no effect. Together, these observations raise the intriguing possibility that P-cadherin could affect the posttranslational events of β1 integrin, and subsequently its activity in ovarian cancer cells.

**Figure 6:**
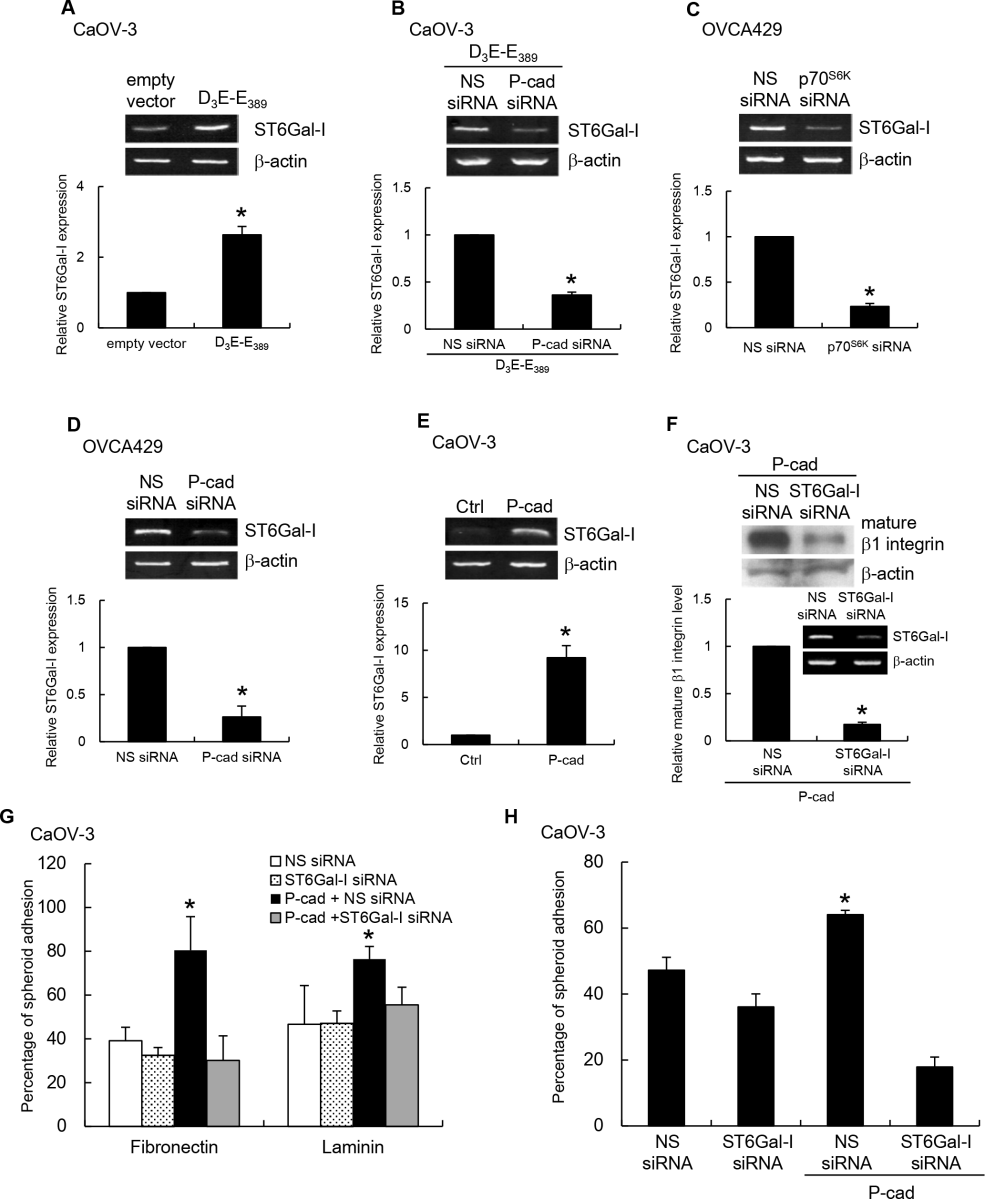
p70^S6K^–P-cadherin signaling regulates β1 integrin maturation through ST6Gal-I CaOV-3 spheroids were transfected with **(A)** constitutively active p70^S6K^ (D_3_E-E_389_) alone or **(B)** cotransfected with nonspecific (NS) siRNA or P-cadherin siRNA. OVCA429 spheroids were transfected with NS siRNA, **(C)** p70^S6K^ siRNA, or **(D)** P-cadherin siRNA. **(E)** CaOV-3 spheroids were stably transfected with P-cadherin. Total RNA was extracted and RT-PCR was carried out with the primer specific to ST6Gal-I. β-actin served as the control. Signal intensity was determined by densitometry and levels of ST6Gal-I were normalized against the β-actin control. CaOV-3 spheroids stably expressing P-cadherin were transfected with NS siRNA or ST6Gal-I siRNA. **(F)** The level of mature form of β1 integrin in whole cell lysates was analyzed by Western blotting. The knockdown efficiency of ST6Gal-I siRNA was analyzed by RT-PCR (insert). In addition, spheroids were collected for the adhesion assay with **(G)** fibronectin, laminin or **(H)** mesothelial cells. The percentage of adherent spheroids was quantified after nonadherent spheroids were removed. Data are expressed as mean ± S.D. *, *P* < 0.05 vs. control, empty vector or NS siRNA expressing spheroids.

N-glycans of β1 integrin are known to have a different carbohydrate compositions after cell transformation [17]. Recent studies suggest that β1 integrin is a substrate for the Golgi glycosyltransferase, ST6Gal-I [18, 19]. To investigate more in detail the regulation of β1 integrin by P-cadherin, we tested the involvement of ST6Gal-I. A strong induction of ST6Gal-1 was observed in p70^S6K^-expressing CaOV-3 spheroids (Figure 6A), which was markedly reduced with P-cadherin siRNA treatment, but nonspecific siRNA treatment had no effect (Figure 6B). In support, knockdown of p70^S6K^ (Figure 6C) or P-cadherin (Figure 6D) in OVCA429 significantly inhibited ST6Gal-I expression, whereas overexpression of P-cadherin in CaOV-3 enhanced ST6Gal-I expression (Figure 6E). The knockdown of ST6Gal-I significantly inhibited the P-cadherin-induced mature β1 integrin expression (Figure 6F), indicating that ST6Gal-I was involved in this process. These data indicate P-cadherin plays a role in the increased expression of ST6Gal-I by p70^S6K^ activation. Using spheroid adhesion assays, we further showed that α2-6 sialylation of β1 integrin was required for the P-cadherin-mediated adhesion to fibronectin, laminin (Figure 6G), and mesothelial cells (Figure 6H).

### P-cadherin-dependent ST6Gal-I induction is mediated by p120 catenin (p120^ctn^)

**Figure 7:**
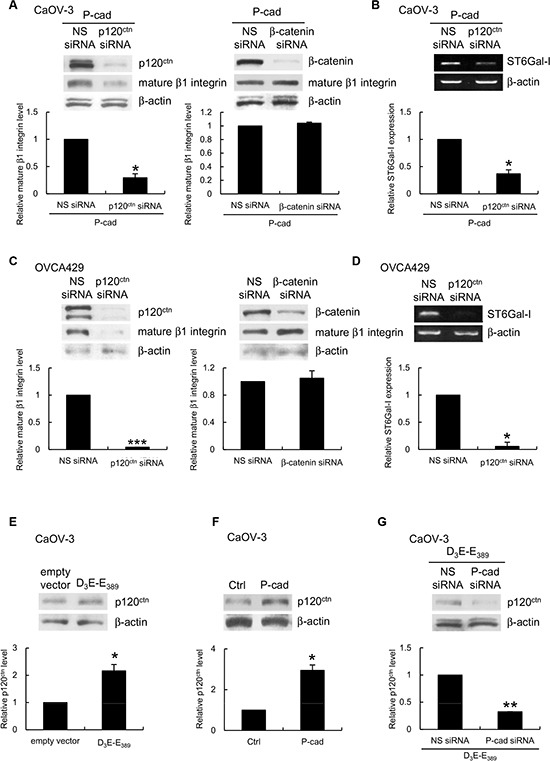
P-cadherin-ST6Gal-I dependent β1 integrin maturation is mediated by p120^ctn^ CaOV-3 spheroids stably expressing P-cadherin or OVCA429 spheroids were transfected with nonspecific (NS) siRNA, p120^ctn^ siRNA, or β-catenin siRNA. **(A, C)** The level of the mature form of β1 integrin in whole cell lysates was analyzed by Western blotting. **(B, D)** Total RNA was extracted and the level of ST6Gal-I was analyzed by RT-PCR. Whole cell lysates of CaOV3 spheroids **(E)** expressing empty vector or constitutively active p70^S6K^ (D_3_E-E_389_), or **(F)** stably expressing P-cadherin, or **(G)** coexpressing D_3_E-E_389_ with NS or P-cadherin siRNA were analyzed for the level of p120^ctn^ by Western blotting. β-actin served as the control for both Western blotting and RT-PCR. Signal intensity was determined by densitometry and levels of the mature form of β1 integrin, ST3Gal-I or p120^ctn^ were normalized against the β-actin control. Data are expressed as mean ± S.D. *, *P* < 0.05; **, *P* < 0.005; ***, P < 0.001 vs. empty vector or NS siRNA expressing spheroids.

To determine how P-cadherin interacts with ST6Gal-I at the molecular level, we evaluated the potential contributions of β-catenin and p120^ctn^, which are associated with the intracellular domain of P-cadherin and are known to affect gene expression. We used siRNAs to knockdown β-catenin and p120^ctn^ expression in P-cadherin-overexpressing CaOV-3 spheroids. Knockdown of p120^ctn^ (Figure 7A, left), but not knockdown of β-catenin (Figure 7A, right), dramatically reduced the expression of mature β1 integrin in P-cadherin-overexpressing CaOV-3 spheroids. Knockdown of p120^ctn^ expression prevented the upregulation of ST6Gal-I in response to P-cadherin (Figure 7B). In OVCA429 spheroids, knockdown of p120^ctn^, but not knockdown of β-catenin, had similar effects on β1 integrin (Figure 7C) and ST6Gal-I (Figure 7D) expression. In addition, we observed an increased level of p120^ctn^ in D_3_E-E_389_-(Figure 7E) and P-cadherin-expressing CaOV-3 spheroids (Figure 7F), but silencing of P-cadherin abolished this (Figure 7G). These results show the P-cadherin-ST6Gal-I interaction was mediated by canonical p120^ctn^ signaling.

### p70^S6K^ inhibition reduces adhesion and peritoneal metastasis

**Figure 8:**
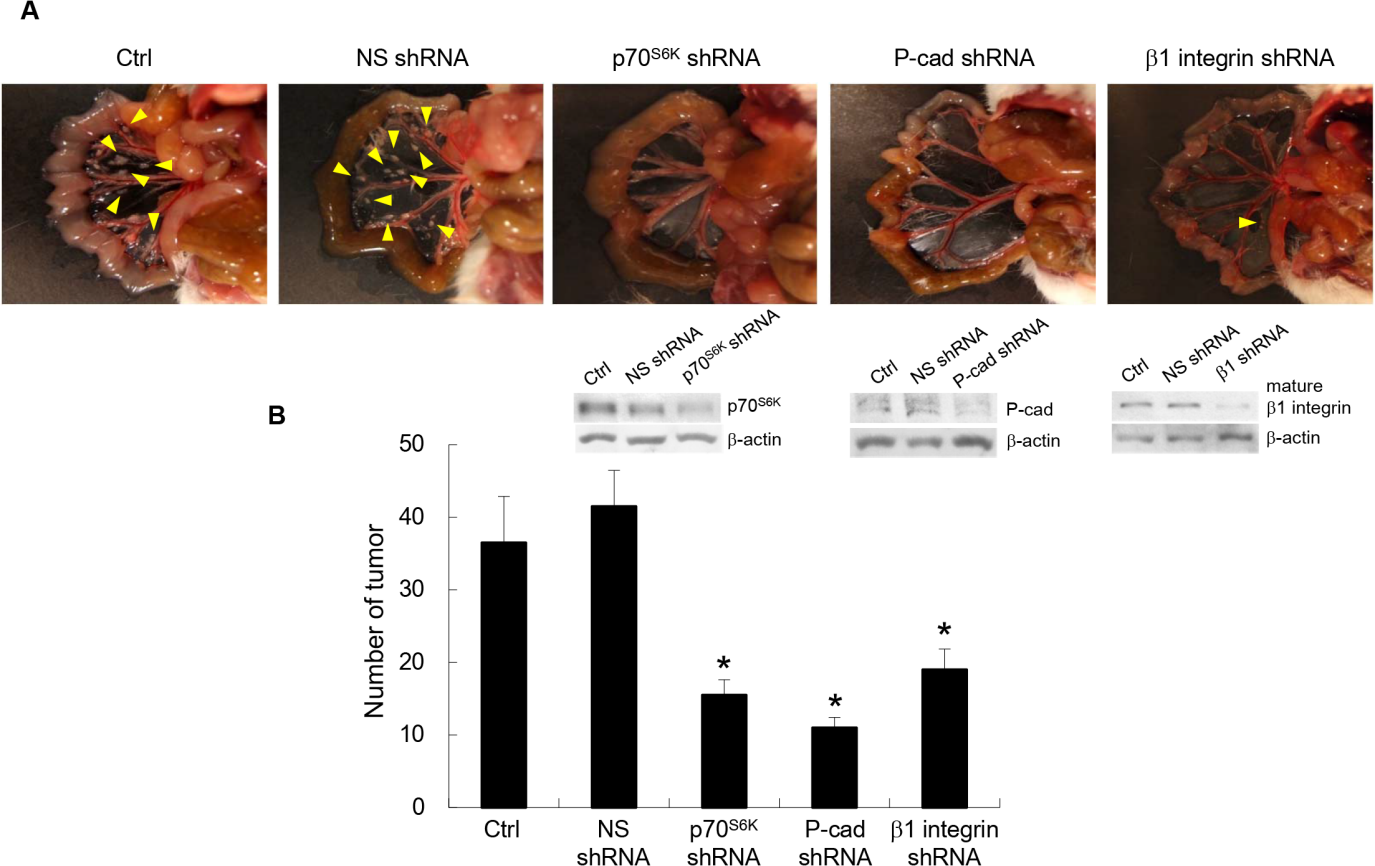
Inhibition of p70^S6K^-P-cadherin-β1 integrin signaling reduces peritoneal metastasis *in vivo* **(A)** Female NOD SCID mice were injected intraperitoneally with 1x10^7^ OVCA429 cells (obtained from spheroid cultures) transduced with nonspecific (NS) shRNA, p70^S6K^ shRNA, P-cadherin shRNA, and β1 integrin shRNA. They were sacrificed 15 weeks after injection. Representative views of the metastases in the peritoneal cavity are shown. Arrows indicate tumor. **(B)** Tumors were counted at autopsy. Data are expressed as mean ± S.D. *, *P* < 0.05 vs. NS shRNA expressing spheroids.

The data so far indicate that β1 integrin is upregulated by P-cadherin upon p70^S6K^ activation and to induce adhesion *in vitro*. We further investigated the interplay between p70^S6K^, P-cadherin, and β1-integrin in the metastatic process in ovarian cancer. We examined the effect of p70^S6K^, P-cadherin, and β1 integrin shRNA on ovarian cancer spheroids growth in the peritoneal cavity using an orthotopic mouse model with advanced ovarian cancer [20]. Control mice or nonspecific shRNA-treated mice showed multiple tumors on the omentum, mesenterium and small bowel (Figure 8). The tumor burden as gauged by the number of disseminated tumor nodules was significantly fewer in mice injected with p70^S6K^ (62.6% inhibition, *P* = 0.024), P-cadherin (73.5% inhibition, *P* = 0.047), and β1 integrin (54.2% inhibition, *P* = 0.021) shRNA-expressing spheroids. These *in vivo* data further support the importance of a p70^S6K^-P-cadherin-β1 integrin signaling axis in ovarian cancer metastasis.

## DISCUSSION

Understanding the early steps of metastasis is crucial to identify novel targets for cancer therapeutics. Peritoneal metastasis is a lethal progression of ovarian cancer, and determining the molecular mechanisms involved is a pressing need. Here for the first time, we provide evidence that p70^S6K^, a key intracellular signaling mediator of multiple growth factors in malignant ascites and is frequently activated in human ovarian cancer, plays a critical role in adhesion of ovarian cancer cells to the peritoneal mesothelium, which is one of the earliest steps in metastasis. This would suggest that p70^S6K^ might be as important in tumor progression as other very early genetic abnormalities, which could be targeted for cancer treatments. These types of treatment would be intriguing because current targeted therapies for ovarian cancer focus on counteracting the progression (e.g. vascular endothelial growth factor or angiogenesis) rather than the initiation of metastasis.

We also demonstrated a new mechanism of action of p70^S6K^ in peritoneal adhesion, which involved P-cadherin and β1 integrin as targets. This observation also expands on our previous findings. We previously detected significant levels of P-cadherin in the metastatic adenocarcinoma in omentum or peritoneum and on mesothelial cells, suggesting that P-cadherin may mediate tumor-mesothelial cell adhesion [21]. This function could be explained by the fact that P-cadherin is the predominant subtype expressed in the peritoneum and by the unique ability of cadherins to engage in homophilic (i.e., between the same cadherins) and heterotypic (i.e., between two different cell types) interactions [22, 23]. The involvement of β1 integrin in ovarian tumorigenesis has also been supported by several studies [24]. Clinically, high β1 integrin expression is indicative of a poor outcome [25]. Treatment with a β1 integrin antibody either before disseminated tumors develop or after solid tumors are established within the peritoneal cavity have been shown to be effective in inhibiting ovarian cancer metastasis [26]. In addition to their involvement in the adhesion of ovarian cancer cells to peritoneal mesothelial cells, it is important to emphasize that both P-cadherin and β1 integrin may activate ‘outside-in signaling’ to stimulate subsequent migration and invasion [27, 28].

The existence of interplay between members of different families of adhesion molecules has been previously suggested in other cellular systems. For example, N-cadherin expression stimulates directional and collective cell migration by activating integrins [29]. Likewise, in squamous carcinoma cells, the signaling pathway between T-cadherin and β1 integrin is important for aggressive invasion and distant metastasis [30]. The biological implication of this crosstalk in ovarian cancer is potentially of high functional significance because this fine-tuned mechanism ensures efficient coordination between adhesion systems, where spatial and temporal coordinated expression and activation of multiple receptors are more important for regulating cell adhesion than the intrinsic specificity of individual receptors [31-33]. In particular, expression of β1 integrin could give ovarian cancer cells a selection advantage that allows them, upon displacement of the mesothelial cells, to attach to the underlying ECM. The expression of P-cadherin induces cell-matrix adhesion, which is consistent with the hypothesis that cadherins are master regulators of cell adhesion and are required for establishing diverse cell junctions [34].

One of the novel findings of the present study is that P-cadherin was shown to be an activator of ST6Gal-I synthesis. In many tissues or cell type, ST6Gal-I is differentially expressed. This suggests that the gene expression of ST6Gal-I could be controlled by specific promoters or corresponding transcriptional factors of the oncogene and tumor suppressor pathways to regulate ST6Gal-I expression [35]. For example, the Ras oncogene, liver-enriched factors HNF-1 and DBP, and transcription factors AP-1 and AP-2 have all been reported to participate in the transcriptional activity of ST6Gal-I [36-38]. This study is the first to demonstrate that ST6Gal-I is a target gene of P-cadherin, adding P-cadherin as a new member of transcriptional modulators of ST6Gal-I.

Our findings provide the first evidence of the crosstalk between P-cadherin and β1 integrin is based on the regulation of β1 integrin activation that occurs at the post-translation modification level. The rapidity with which β1 integrin becomes activated could be the mechanism by which ovarian cancer cells can specifically and rapidly respond to environmental changes. Although mechanisms have not been fully elucidated, α2-6 sialylation could modify integrin-dependent cell responses by changing receptor conformation or by indirect mechanisms with other membrane-associated proteins [17]. Of interest, ST6Gal-I is overexpressed in many types of cancers including ovarian cancer and this upregulation correlates with increased metastatic potential and poor prognosis [39-41].

The experiments in this study examined spheroids that closely mimic conditions *in vivo* and could be of clinical importance. Increasing evidence indicates that ovarian carcinoma cells cultured as spheroids exhibit different proliferative and adhesive properties compared to monolayer cultures [42, 43]. Our results suggest that targeting p70^S6K^ could provide the best approach to inhibiting the progression of ovarian cancer in its initial stage, including ovarian carcinomas that may be localized in the ovary but have malignant ascites or positive peritoneal washings, and also that have not been completely removed during surgery at the microscopic level.

Many inhibitors that target the mTOR/p70^S6K^ pathway have been developed and some are currently being tested in clinical trials. For example, RAD001 (Everolimus) has been shown to be effective in delaying tumor progression and prolonging survival in both xenografts and transgenic mice models of ovarian cancer [44, 45]. CCI-779 (Temsirolimus) tested in patients with malignant ovarian cancer resulted in the apparent reduction of ascites and decreased peritoneal dissemination of tumor cells [46]. NVP-BEZ235 could effectively inhibit growth of ovarian cancer cells even with platinum resistance and could prolong survival of mice with intraperitoneal ovarian tumors [47]. Furthermore, rapamycin and several of its analogs have been demonstrated to have synergistic cytotoxic effect with other chemotherapeutic agents on ovarian cancer [45, 48, 49]. Recent evidence suggests that mTOR/p70^S6K^ signaling is overactivated in p53 deficient or mutant cells [50-52]. Alterations in p53 are common in advanced ovarian cancer [53]. The diverse types of p53 mutants make it difficult to develop a versatile p53-reactivating drug, but targeting the mTOR/p70^S6K^ pathway could be an alternative strategy. Detailed tumor intervention studies and survival analyses on ovarian cancer treatments targeting this pathway certainly merit further investigation.

In summary, our study provides new findings show that p70^S6K^ increases peritoneal adhesion and dissemination of ovarian cancer. Our observation that activation of P-cadherin/β1 integrin crosstalk promotes peritoneal metastasis of ovarian cancer indicates this signaling pathway could play a role in ovarian cancer malignancy. Because patient's prognosis depends on the tumor residue, it is critical to understand the molecular events that regulate metastasis. Our results should be a step in this direction and provide a strong rationale to explore the targeting of p70^S6K^ in the context of novel strategies against ovarian cancer dissemination.

## MATERIALS AND METHODS

### Cell lines and cell culture

Human ovarian CaOV-3 (a gift from Dr. Nelly Auersperg, University of British Columbia, Vancouver, B. C., Canada), OV-90 and OVCA429 cancer cell lines were maintained in M199:MCDB105 supplemented with 5% fetal bovine serum (Hyclone, Logan, UT, USA) at 37^o^C under 5% CO_2_. To generate spheroids, cells were dissociated by trypsinization and plated on non-adhesive culture dishes as described [54]. Spheroids were collected for experiments after 36 to 48 h. Each spheroid was about 100 μm in diameter containing about 500-1,000 cells. Human peritoneal tissue was obtained from patients undergoing surgery for benign conditions. Mesothelial cells were isolated as described [55], and were cultured at 37^o^C for 2 to 3 days until a monolayer of polygonal cells had grown.

### siRNA, shRNA, and cDNA plasmids

Specific siRNA oligonucleotides against p70^S6K^ (5′-GACAAAAUCCUCAAAUGUA-3′), P-cadherin (5′-GAGGGUGUCUUCGCUGUAG-3′), β1 integrin (5′-CCACAGACA UUUACAUUAA-3′), ST6Gal-I (5′-ACUCAGAUAUCCCAAAGUG-3′), p120-catenin (5′-UAGCUGACCUCCUGACUAA-3′), β-catenin (5′-AAGUCCUGUAUGAGUGGG AAC-3′), and nonspecific duplex oligo (5′-GGCTACGTCCAGGAGCGCA-3′) were obtained from Dharmacon (Lafayette, CO, USA). Transfection was performed using siLentFect (Bio-Rad, Hercules, CA, USA) according to the manufacturer's procedures. To stably attenuate expression of p70^S6K^, β1 integrin, and P-cadherin, we infected cells with lentivirus containing p70^S6K^ shRNA, β1 integrin shRNA or P-cadherin shRNA, or nonspecific shRNA as a control. Lentivrus-infected cells were selected for 72 hours in medium containing 1 μg/ml puromycin (Invitrogen, San Diego, CA, USA). To ectopically express active p70^S6K^ (D_3_E-E_389_) in CaOV-3 cells, we used a myc-tagged cDNA encoding the constitutively active p70^S6K^ (generously given by Dr. George Thomas, Genome Research Institute, University of Cincinnati, OH, USA).

### Cancer spheroid adhesion assays

For measurement of adhesion to human peritoneal mesothelial cells, fluorescent-labeled spheroids were overlaid onto a monolayer of confluent primary human mesothelial cells coated on a 24-well plate and allowed to attach for 1 to 5 h before counting. For measurement of adhesion to ECM components, plates were first coated with fibronectin, laminin, vitronectin and collagen I (10 μg/ml) according to the manufacturer's instructions (BD Biosciences, San Jose, CA, USA), spheroids were then added and allowed to attach for 1 to 5 h before counting. For the inhibition experiments, spheroids were pretreated with a neutralizing antibody to β1 integrin (clone JB1A; 1:100) (Chemicon, Chandlers Ford, UK) at 37^o^C for 30 min prior to incubation with mesothelial cells or the different ECM components. The total number of spheroids in each well was counted prior to removal of nonadherent spheroids. Nonadherent spheroids were removed by gentle washing with PBS. The remaining adhered spheroids were fixed and counted. The percentage of adhered spheroid was calculated by dividing the number of spheroids adhering on the mesothelium or ECM components by the total number of spheroids in each well before washing.

### Reverse transcription-PCR

Total RNA was isolated using Trizol (Invitrogen) according to the manufacturer's instructions. Total RNA (500 ng) was reverse transcribed using a First-strand Reverse Transcription Kit (Invitrogen), and PCR was performed with a set of primers: ST6Gal-I, sense 5′-TATCGTAAGCTGCACCCCAATC-3′, anti-sense 5′-TTAGCAGTGAATG GTCCGGAAG-3′; and β-actin, sense 5′-TCACCGAGGCCCCTCTGAACCCTA-3′, anti-sense 5′-GGCAGTAATCTCCTTCTGCATCCT-3′. The number of amplification cycles, during which the PCR product formation was limited by the template concentration was determined in pilot experiments. The mRNA levels of target genes were normalized to that of β-actin.

### Western blotting

Cells were harvested in SDS sample buffer (65 mM Tris-Cl pH 6.8, 2% SDS, 10% glycerol) supplemented with protease inhibitors (1 μg/ml aprotinin, 1 μg/ml leupeptin, 1 μg/ml pepstatin A, 1 mM phenylmethyl sulfonyl), 1 mM sodium orthovanadate and 1 mM sodium fluoride) and quantified using a DC protein assay kit (Bio-Rad). Equal amounts of protein were separated on SDS-polyacrylamide gels and transferred to nitrocellulose membrane. Membranes were then blocked and probed with the following primary antibodies: anti-c-myc (clone 9E10; 1:1,500), anti-phospho-p70^S6K^ (Thr389; 1:1,000), anti-p70^S6K^ (1:1,000), anti-phospho-S6 (Ser235/236; 1:1,000), anti-S6 (1:1,000) (Cell Signaling, Beverley, MA, USA), anti-β1 integrin (1:1,000) (Chemicon, Chandlers Ford, UK), anti-β-catenin (1:2,000), anti-p120^ctn^ (1:1,000), anti-P-cadherin (1:1000) (Transduction Laboratories, Lexington, KY, USA) and anti-β-actin (1:2,000) (Sigma, St Louis, MO, USA). Western blot membranes were visualized using an enhanced chemiluminescent substrate for detection of horseradish peroxidase (Amersham, Little Chalfont, UK).

### Mouse model of ovarian cancer metastasis

4-6 week-old female NOD SCID mice (Charles River Laboratories, Wilmington, MA) were used (n = 3 per group), and the experiment was conducted twice. 1x10^7^ cells from the spheroid cultures were suspended in 0.1 ml Hanks' Balanced salt solution and were injected intraperitoneally into the peritoneal cavity. The mice were sacrificed and the numbers of disseminated tumor nodules within the peritoneal cavity were counted. All animal studies were performed using protocols approved by the University of Hong Kong Institutional Animal Care and Use Committee.

### Statistical analyses

*P* values were based upon Student's t test using GraphPad Prism (San Diego, CA, USA). *P* < 0.05 was considered to be statistically significant on the basis of at least three independent sets of experiments.

## References

[1] Siegel R, Naishadham D, Jemal A (2012). Cancer statistics, 2012. CA Cancer J Clin.

[2] Ip CK, Wong AS (2012). Exploiting p70 S6 kinase as a target for ovarian cancer. Expert Opin Ther Targets.

[3] Altomare DA, Wang HQ, Skele KL, De Rienzo A, Klein-Szanto AJ, Godwin AK, Testa JR (2004). AKT and mTOR phosphorylation is frequently detected in ovarian cancer and can be targeted to disrupt ovarian tumor cell growth. Oncogene.

[4] Zhou HY, Wong AST (2006). Activation of p70(S6K) induces expression of matrix metalloproteinase 9 associated with hepatocyte growth factor-mediated invasion in human ovarian cancer cells. Endocrinology.

[5] Castellvi J, Garcia A, Rojo F, Ruiz-Marcellan C, Gil A, Baselga J, Cajal SRY (2006). Phosphorylated 4E binding protein 1: A hallmark of cell signaling that correlates with survival in ovarian cancer. Cancer.

[6] Pon YL, Zhou HY, Cheung ANY, Ngan HYS, Wong AST (2008). p70 S6 kinase promotes epithelial to mesenchymal transition through Snail induction in ovarian cancer cells. Cancer Res.

[7] Hynes RO (2002). Integrins: bidirectional, allosteric signaling machines. Cell.

[8] Sundfeldt K (2003). Cell-cell adhesion in the normal ovary and ovarian tumors of epithelial origin; an exception to the rule. Mol Cell Endocrinol.

[9] Elmasri WM, Casagrande G, Hoskins E, Kimm D, Kohn EC (2009). Cell adhesion in ovarian cancer. Cancer Treat Res.

[10] Sowter HM, Corps AN, Smith SK (1999). Hepatocyte growth factor (HGF) in ovarian epithelial tumour fluids stimulates the migration of ovarian carcinoma cells. Int J Cancer.

[11] Sawada K, Radjabi AR, Shinomiya N, Kistner E, Kenny H, Becker AR, Turkyilmaz MA, Salgia R, Yamada SD, Vande Woude GF, Tretiakova MS, Lengyel E (2007). c-Met overexpression is a prognostic factor in ovarian cancer and an effective target for inhibition of peritoneal dissemination and invasion. Cancer Res.

[12] Wong AS, Pelech SL, Woo MM, Yim G, Rosen B, Ehlen T, Leung PC, Auersperg N (2001). Coexpression of hepatocyte growth factor-Met: an early step in ovarian carcinogenesis?. Oncogene.

[13] Wong AST, Roskelley CD, Pelech S, Miller D, Leung PCK, Auersperg N (2004). Progressive changes in Met-dependent signaling in a human ovarian surface epithelial model of malignant transformation. Exp Cell Res.

[14] Moser TL, Pizzo SV, Bafetti LM, Fishman DA, Stack MS (1996). Evidence for preferential adhesion of ovarian epithelial carcinoma cells to type I collagen mediated by the alpha2beta1 integrin. Int J Cancer.

[15] Wilson AP (1989). Mesothelial cells stimulate the anchorage-independent growth of human ovarian tumour cells. Br J Cancer.

[16] Tozer EC, Hughes PE, Loftus JC (1996). Ligand binding and affinity modulation of integrins. Biochem Cell Biol.

[17] Bellis SL (2004). Variant glycosylation: an underappreciated regulatory mechanism for beta1 integrins. Biochim Biophys Acta.

[18] Semel AC, Seales EC, Singhal A, Eklund EA, Colley KJ, Bellis SL (2002). Hyposialylation of integrins stimulates the activity of myeloid fibronectin receptors. J Biol Chem.

[19] Seales EC, Jurado GA, Singhal A, Bellis SL (2003). Ras oncogene directs expression of a differentially sialylated, functionally altered beta1 integrin. Oncogene.

[20] Shaw TJ, Senterman MK, Dawson K, Crane CA, Vanderhyden BC (2004). Characterization of intraperitoneal, orthotopic, and metastatic xenograft models of human ovarian cancer. Mol Ther.

[21] Cheung LW, Mak AS, Cheung AN, Ngan HY, Leung PC, Wong AS (2011). P-cadherin cooperates with insulin-like growth factor-1 receptor to promote metastatic signaling of gonadotropin-releasing hormone in ovarian cancer via p120 catenin. Oncogene.

[22] Gumbiner BM (2005). Regulation of cadherin-mediated adhesion in morphogenesis. Nat Rev Mol Cell Biol.

[23] Takeichi M (1995). Morphogenetic roles of classic cadherins. Curr Opin Cell Biol.

[24] Burleson KM, Hansen LK, Skubitz AP (2004). Ovarian carcinoma spheroids disaggregate on type I collagen and invade live human mesothelial cell monolayers. Clin Exp Metastasis.

[25] Muller-Klingspor V, Hefler L, Obermair A, Kaider A, Breitenecker G, Leodolte S, Kohlberger P (2001). Prognostic value of beta1-integrin (=CD29) in serous adenocarcinomas of the ovary. Anticancer Res.

[26] Mitra AK, Sawada K, Tiwari P, Mui K, Gwin K, Lengyel E (2011). Ligand-independent activation of c-Met by fibronectin and alpha(5)beta(1)-integrin regulates ovarian cancer invasion and metastasis. Oncogene.

[27] Cheung LW, Leung PC, Wong AS (2010). Cadherin switching and activation of p120 catenin signaling are mediators of gonadotropin-releasing hormone to promote tumor cell migration and invasion in ovarian cancer. Oncogene.

[28] Buczek-Thomas JA, Chen N, Hasan T (1998). Integrin-mediated adhesion and signalling in ovarian cancer cells. Cell Signal.

[29] Ouyang M, Lu S, Kim T, Chen CE, Seong J, Leckband DE, Wang F, Reynolds AB, Schwartz MA, Wang Y (2013). N-cadherin regulates spatially polarized signals through distinct p120ctn and beta-catenin-dependent signalling pathways. Nat Commun.

[30] Mukoyama Y, Utani A, Matsui S, Zhou S, Miyachi Y, Matsuyoshi N (2007). T-cadherin enhances cell-matrix adhesiveness by regulating beta1 integrin trafficking in cutaneous squamous carcinoma cells. Genes Cells.

[31] Marsden M, DeSimone DW (2003). Integrin-ECM interactions regulate cadherin-dependent cell adhesion and are required for convergent extension in Xenopus. Curr Biol.

[32] Martinez-Rico C, Pincet F, Thiery JP, Dufour S (2010). Integrins stimulate E-cadherin-mediated intercellular adhesion by regulating Src-kinase activation and actomyosin contractility. J Cell Sci.

[33] Yano H, Mazaki Y, Kurokawa K, Hanks SK, Matsuda M, Sabe H (2004). Roles played by a subset of integrin signaling molecules in cadherin-based cell-cell adhesion. J Cell Biol.

[34] Niessen CM, Gottardi CJ (2008). Molecular components of the adherens junction. Biochim Biophys Acta.

[35] Dall'Olio F (2000). The sialyl-alpha2,6-lactosaminyl-structure: biosynthesis and functional role. Glycoconj J.

[36] Le Marer N, Laudet V, Svensson EC, Cazlaris H, Van Hille B, Lagrou C, Stehelin D, Montreuil J, Verbert A, Delannoy P (1992). The c-Ha-ras oncogene induces increased expression of beta-galactoside alpha-2, 6-sialyltransferase in rat fibroblast (FR3T3) cells. Glycobiology.

[37] Svensson EC, Soreghan B, Paulson JC (1990). Organization of the beta-galactoside alpha 2,6-sialyltransferase gene. Evidence for the transcriptional regulation of terminal glycosylation. J Biol Chem.

[38] Vandamme V, Cazlaris H, Le Marer N, Laudet V, Lagrou C, Verbert A, Delannoy P (1992). Comparison of sialyl- and alpha-1,3-galactosyltransferase activity in NIH3T3 cells transformed with ras oncogene: increased beta-galactoside alpha-2,6-sialyltransferase. Biochimie.

[39] Christie DR, Shaikh FM, Lucas JAt, Lucas JA, Bellis SL (2008). ST6Gal-I expression in ovarian cancer cells promotes an invasive phenotype by altering integrin glycosylation and function. J Ovarian Res.

[40] Dall'Olio F, Chiricolo M (2001). Sialyltransferases in cancer. Glycoconj J.

[41] Schultz MJ, Swindall AF, Wright JW, Sztul ES, Landen CN, Bellis SL (2013). ST6Gal-I sialyltransferase confers cisplatin resistance in ovarian tumor cells. J Ovarian Res.

[42] Tang MK, Zhou HY, Yam JW, Wong AS (2010). c-Met overexpression contributes to the acquired apoptotic resistance of nonadherent ovarian cancer cells through a cross talk mediated by phosphatidylinositol 3-kinase and extracellular signal-regulated kinase 1/2. Neoplasia.

[43] Zietarska M, Maugard CM, Filali-Mouhim A, Alam-Fahmy M, Tonin PN, Provencher DM, Mes-Masson AM (2007). Molecular description of a 3D in vitro model for the study of epithelial ovarian cancer (EOC). Mol Carcinog.

[44] Mabuchi S, Altomare DA, Cheung M, Zhang L, Poulikakos PI, Hensley HH, Schilder RJ, Ozols RF, Testa JR (2007). RAD001 inhibits human ovarian cancer cell proliferation, enhances cisplatin-induced apoptosis, and prolongs survival in an ovarian cancer model. Clinical cancer research: an official journal of the American Association for Cancer Research.

[45] Mabuchi S, Altomare DA, Connolly DC, Klein-Szanto A, Litwin S, Hoelzle MK, Hensley HH, Hamilton TC, Testa JR (2007). RAD001 (Everolimus) delays tumor onset and progression in a transgenic mouse model of ovarian cancer. Cancer Res.

[46] Takano M, Kikuchi Y, Kudoh K, Goto T, Furuya K, Kikuchi R, Kita T, Fujiwara K, Shiozawa T, Aoki D (2011). Weekly administration of temsirolimus for heavily pretreated patients with clear cell carcinoma of the ovary: a report of six cases. International journal of clinical oncology.

[47] Santiskulvong C, Konecny GE, Fekete M, Chen KY, Karam A, Mulholland D, Eng C, Wu H, Song M, Dorigo O (2011). Dual targeting of phosphoinositide 3-kinase and mammalian target of rapamycin using NVP-BEZ235 as a novel therapeutic approach in human ovarian carcinoma. Clinical cancer research: an official journal of the American Association for Cancer Research.

[48] Huynh H, Teo CC, Soo KC (2007). Bevacizumab and rapamycin inhibit tumor growth in peritoneal model of human ovarian cancer. Molecular cancer therapeutics.

[49] Piha-Paul SA, Wheler JJ, Fu S, Levenback C, Lu K, Falchook GS, Naing A, Hong DS, Tsimberidou AM, Kurzrock R (2014). Advanced gynecologic malignancies treated with a combination of the VEGF inhibitor bevacizumab and the mTOR inhibitor temsirolimus. Oncotarget.

[50] Comas M, Toshkov I, Kuropatwinski KK, Chernova OB, Polinsky A, Blagosklonny MV, Gudkov AV, Antoch MP (2012). New nanoformulation of rapamycin Rapatar extends lifespan in homozygous p53−/− mice by delaying carcinogenesis. Aging.

[51] Komarova EA, Antoch MP, Novototskaya LR, Chernova OB, Paszkiewicz G, Leontieva OV, Blagosklonny MV, Gudkov AV (2012). Rapamycin extends lifespan and delays tumorigenesis in heterozygous p53+/− mice. Aging.

[52] Leontieva OV, Novototskaya LR, Paszkiewicz GM, Komarova EA, Gudkov AV, Blagosklonny MV (2013). Dysregulation of the mTOR pathway in p53-deficient mice. Cancer biology & therapy.

[53] Romero I, Bast RC (2012). Minireview: human ovarian cancer: biology, current management, and paths to personalizing therapy. Endocrinology.

[54] Iwanicki MP, Davidowitz RA, Ng MR, Besser A, Muranen T, Merritt M, Danuser G, Ince TA, Brugge JS (2011). Ovarian cancer spheroids use myosin-generated force to clear the mesothelium. Cancer Discov.

[55] Yung S, Li FK, Chan TM (2006). Peritoneal mesothelial cell culture and biology. Perit Dial Int.

